# Understanding the research advances on lumpy skin disease: A comprehensive literature review of experimental evidence

**DOI:** 10.3389/fmicb.2022.1065894

**Published:** 2022-11-28

**Authors:** Zhengji Liang, Kaishen Yao, Shasha Wang, Juanbin Yin, Xiaoqin Ma, Xiangping Yin, Xiangwei Wang, Yuefeng Sun

**Affiliations:** State Key Laboratory of Veterinary Etiological Biology, College of Veterinary Medicine, Lanzhou University, Lanzhou Veterinary Research Institute, Chinese Academy of Agricultural Sciences, Lanzhou, China

**Keywords:** lumpy skin disease, lumpy skin disease virus, epidemiology, vaccine, etiology, diagnosis

## Abstract

Lumpy skin disease is caused by lumpy skin disease virus (LSDV), which can induce cattle with high fever and extensive nodules on the mucosa or the scarfskin, seriously influencing the cattle industry development and international import and export trade. Since 2013, the disease has spread rapidly and widely throughout the Russia and Asia. In the past few decades, progress has been made in the study of LSDV. It is mainly transmitted by blood-sucking insects, and various modes of transmission with distinct seasonality. Figuring out how the virus spreads will help eradicate LSDV at its source. In the event of an outbreak, selecting the most effective vaccine to block and eliminate the threat posed by LSDV in a timely manner is the main choice for farmers and authorities. At present, a variety of vaccines for LSDV have been developed. The available vaccine products vary in quality, protection rate, safety and side effects. Early detection of LSDV can help reduce the cost of disease. In addition, because LSDV has a huge genome, it is currently also used as a vaccine carrier, forming a new complex with other viral genes through homologous recombination. The vaccine prepared based on this can have a certain preventive effect on many kinds of diseases. Clinical detection of disease including nucleic acid and antigen level. Each method varies in convenience, accuracy, cost, time and complexity of equipment. This article reviews our current understanding of the mode of transmission of LSDV and advances in vaccine types and detection methods, providing a background for further research into various aspects of LSDV in the future.

## Introduction

Lumpy skin disease (LSD) is an emerging viral transboundary disease which can spread beyond the outbreak area and become epidemic (Jordi et al., 2018; K, 2014; K et al., 2021). The most common clinical symptoms are nodular lesions on the surface of the skin and mucous membranes. Skin nodule lesions often appear on the outside of infected cattle, such as head, neck, back, perineum, breast, and other areas of the cattle (Molla et al., 2017). The affected cattle have varying degrees of edema and lameness in their legs (Salib and Osman, 2011). *In vivo*, they often present with mucosal ulcerations high fever and enlarged lymph nodes (Moyer et al., 2000). It is often manifested as mucopurulent nasal discharge (Lubinga et al., 2015; Elhaig et al., 2017). But this is not the characteristic clinical symptom of LSD. Although a large majority of the affected cattle could recover after a long period of illness, they will have long-term symptoms of mastitis, pneumonia, and deep holes in the hide (Selim et al., 2021a). As it is a fulminating infectious disease, the World Organization for Animal Health (OIE) stipulates LSD is the communicable disease that must be reported. LSDV can spread in many ways, such as indirect contact transmission between animals through vectors, lactation spread, blooding feeding insects, semen spread and iatrogenic transmission (Weiss, 1968; Carn and Kitching, 1995; Mullen and Durden, 2002; Annandale et al., 2010; European Food Safety Authority, 2018). Some researchers have conducted experiments to confirm that the disease is difficult to spread through direct contact (Carn and Kitching, 1995; Magori-Cohen et al., 2012; Mulatu and Feyisa, 2018). LSD was first reported in Zambia, South Africa in 1929 (Rhodesia, 1932). During the past decades, LSDV has spread widely and rapidly throughout the North Africa, Middle Eastern, Asia and other areas of the world, seriously influence the development of the cattle and water buffalo industry (Rgbe, 2014). Among them, the cattle with fine-skinned such as Holstein-Friesian and Jersey are the most susceptible to the virus. However, thick-skinned Bos indicus breeds including the Afrikander show less severe signs of the disease (Coetzer, 2004).

However, there are fewer effective preventive measures for LSD. Restricting the movement of the sick cattle, quarantine, sacrifice the cattle infected with LSDV are heavily recommended (Wolff et al., 2020b). Control and prevention of LSD in the countries like Albania, Bulgaria, Greece, Montenegro, FYROM, Serbia, Ethiopia relies extremely on vaccination (Gari et al., 2015; Klement et al., 2020). Because the LSDV has an intricate immune escape mechanism, no safe and efficient vaccines have been developed for this disease till now. Sheeppox virus (SPPV) and goatpox virus (GTPV) have antigenic homology and cross protection with LSDV; therefore, the vaccines of these two viruses can be used to prevented the LSD. Inevitably, the above two vaccines may have some potential risks because they are live-attenuated vaccines that derived from strains isolated in the field (Tuppurainen et al., 2014; Liu L. et al., 2021a). It is not recommended to use in the disease-free areas.

The diagnostic measures for LSD are mainly aimed at its nucleic acid sequence or corresponding antigen and antibody (Ireland and Binepal, 1998). The accuracy of each diagnostic method varies in a variety of occasions.

The aim of this study is to summarize the research progress of LSDV transmission modes, the types of vaccines used, and detection methods, and to sort out the characteristics of each vaccine and detection method. It will provide a reference for cutting off the spread of diseases, research on safe and efficient vaccines and the development of efficient and fast detection methods.

## Etiology

**Figure 1:**
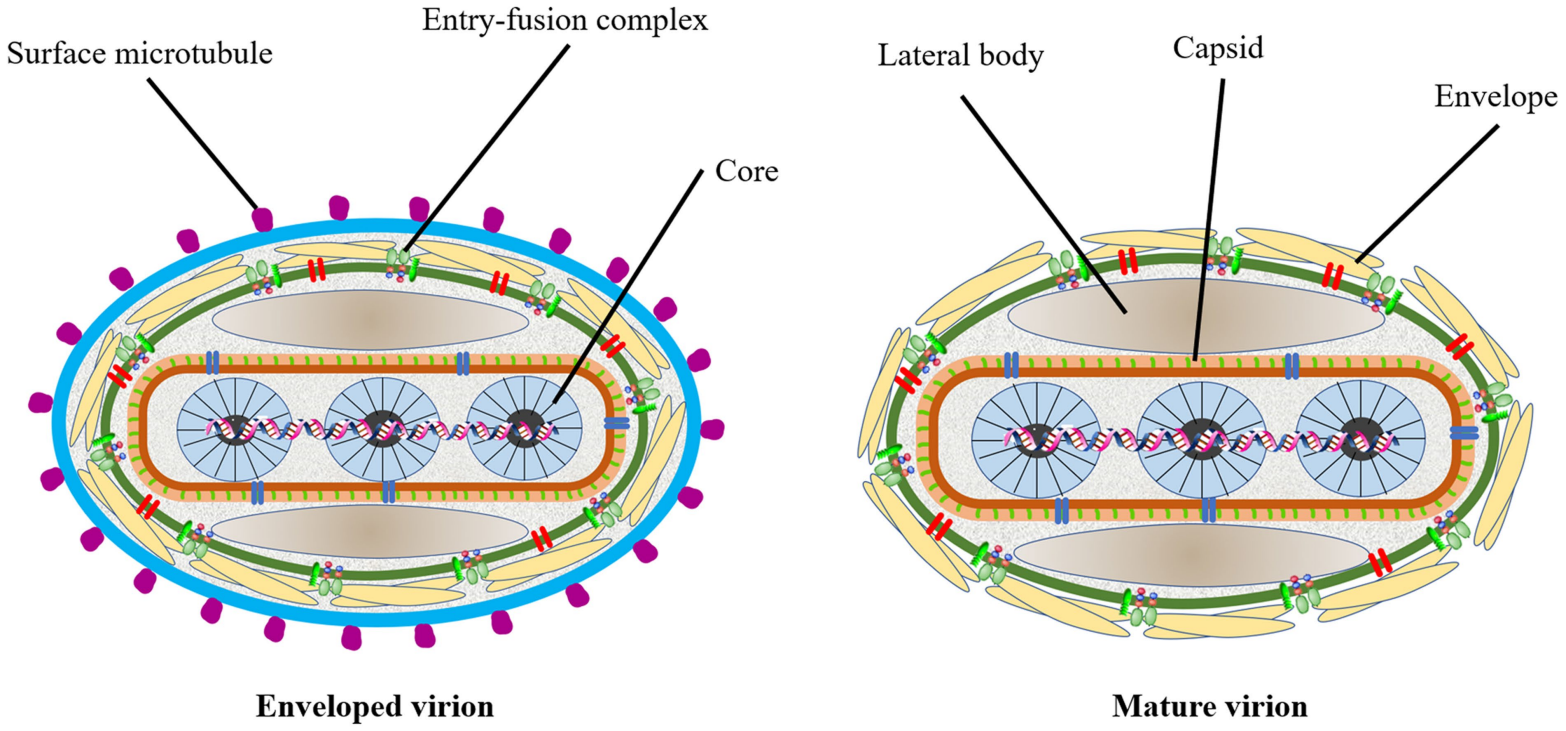
The prediction diagram of LSDV structure mode. Mature virion of LSDV (MV), sometimes mature virion is surrounded by a lipid membrane derived from the endoplasmic reticulum (EV). The surface of the virus is envelope, which contains some entry-fusion complex. The virus contains lateral body, capsid and core. The surface of the EV has many surface microtubules.

LSD is a viral contagious cattle disease caused by Lumpy skin disease virus (LSDV; Murphy et al., 2008). The virus is a large linear double-stranded DNA genomes of 151 kb and belongs to one of the *Capripoxvirus* genus, subfamily *Chordopoxvirniae*, family *Poxviridae* (Tulman et al., 2001; Bhanuprakash et al., 2006; Moss, 2007; K, 2014). Viruses of the *Poxviridae* family are very similar in morphological characteristics (Mcfadden, 2005). Since the researchers have not yet resolved the particle pattern diagram of LSDV, we draw the prediction diagram of LSDV structural pattern based on the pattern diagram of poxvirus for reference (Figure 1). The *Capripoxvirus* genus consists of SPPV, GTPV and LSDV (Tulman et al., 2001; Zare, 2010). These three viruses could cause transboundary disease with serious consequences among the ruminants, causing a major threat to the global animal husbandry (Sprygin et al., 2018a). They all have their own specific natural reservoir. The main hosts of the first two viruses are sheep and goat, while the LSDV mainly affects the cattle and water buffalo (Afonso et al., 2012; Fagbo et al., 2014; Lefkowitz et al., 2018). In addition, LSDV can also infect giraffes, impalas, and wildebeest (Young et al., 1970; Dao et al., 2022). *Capripoxvirus* genus is the most harmfully significant in the *Poxviridae* family affecting domestic ruminants in Africa and Asia. LSDV has more than 97% nucleotide sequence homology with GTPV (Gershon et al., 1989a; Upton, 2004). It is generally acknowledged that, the original pox virus may have originated from one or more basic species. They adapted by spreading the disease among the different kind of susceptible animals (Seet et al., 2003; Sohier et al., 2019). Homologous recombination is the key towards the evolution of the virion. A lot of viruses may have evolved from a common ancestor through genetic recombination within the virus itself to expand their host range and virulence (Gershon et al., 1989b). As a result, poxviruses of various animals were formed. The morphology, structure, biochemistry, and antigenicity of mammalian poxviruses in each genus are similar with each other. After infecting cells with LSDV in the 1960s, Alexander et al. (1957) and Plowright and Witcomb (1959) observed that the inclusion bodies produced in the cytoplasm were highly similar to those produced by other members of the *Poxviridae* family. Later, Munz and Owen (1966) observed that the morphological structure of LSDV and vaccinia virus was also very similar under the electron microscope. In recent years, researchers have observed the appearance of LSDV under the electron microscope, which is indeed similar to the appearance of other virus members in the *Poxviridae* that have been published (Sanz-Bernardo et al., 2020). The length of the virus genome is 151 kb, which consists of a central coding area and a 2.4 kb inverted terminal repeat sequence on both wings. According to the scientific prediction, LSDV has 156 putative genes. It has nine more genes than the other two viruses in the genus. Its morphological characteristics are similar to poxvirus, about 350 nanometers in length and 300 nanometers in width, with envelopes, but no clotting activity. This virus can be proliferated in primary cells, such as lamb and calf kidney or testicular cells, sheep embryonic kidney and lung cells, and chicken embryo fibroblasts. It also can multiply in Madin-Darby bovine kidney cells and baby hamster kidney cells (BHK-21), but the pathological changes are slower. Recent investigations have found that LSDV can hardly proliferate in African green monkey kidney (Vero) cells (Wang et al., 2022). Kumar et al. (2021) treated Vero cells with the product of virus amplification from primary goat kidney (PGK) cells, and it could obtain higher viral titer only after adapting to LSDV and continuing to pass generations. Higher virus titers can be produced in Vero which are cell adapted LSDV (Kumar et al., 2021). This also has new implications for the production of vaccines. In some experiments that need to ensure virulence stability, for example, the construction of recombinant viruses, we choose to perform in Vero cells. LSDV can proliferate on the chorioallantoic membrane of chicken embryo and cause acne-spots, and the virus does not cause the death of the embryo (Black et al., 1986; Upton, 2004; Kumar et al., 2021). The next year, Chinese scientists found that LSDV could produce higher viral titers in primary cattle testicular cells (Wang et al., 2022). When the wild-type virus was attenuated to prepare the vaccine strains, the state of the chicken embryo can be observed as a reference. Because it is a double stranded DNA virus, it has a certain thermal stability. Research shows that LSDV can be completely inactivated at 56°C, making it lose its infectivity (Wolff et al., 2020a).

## Epidemiology

### Geographical distribution of LSD

**Figure 2:**
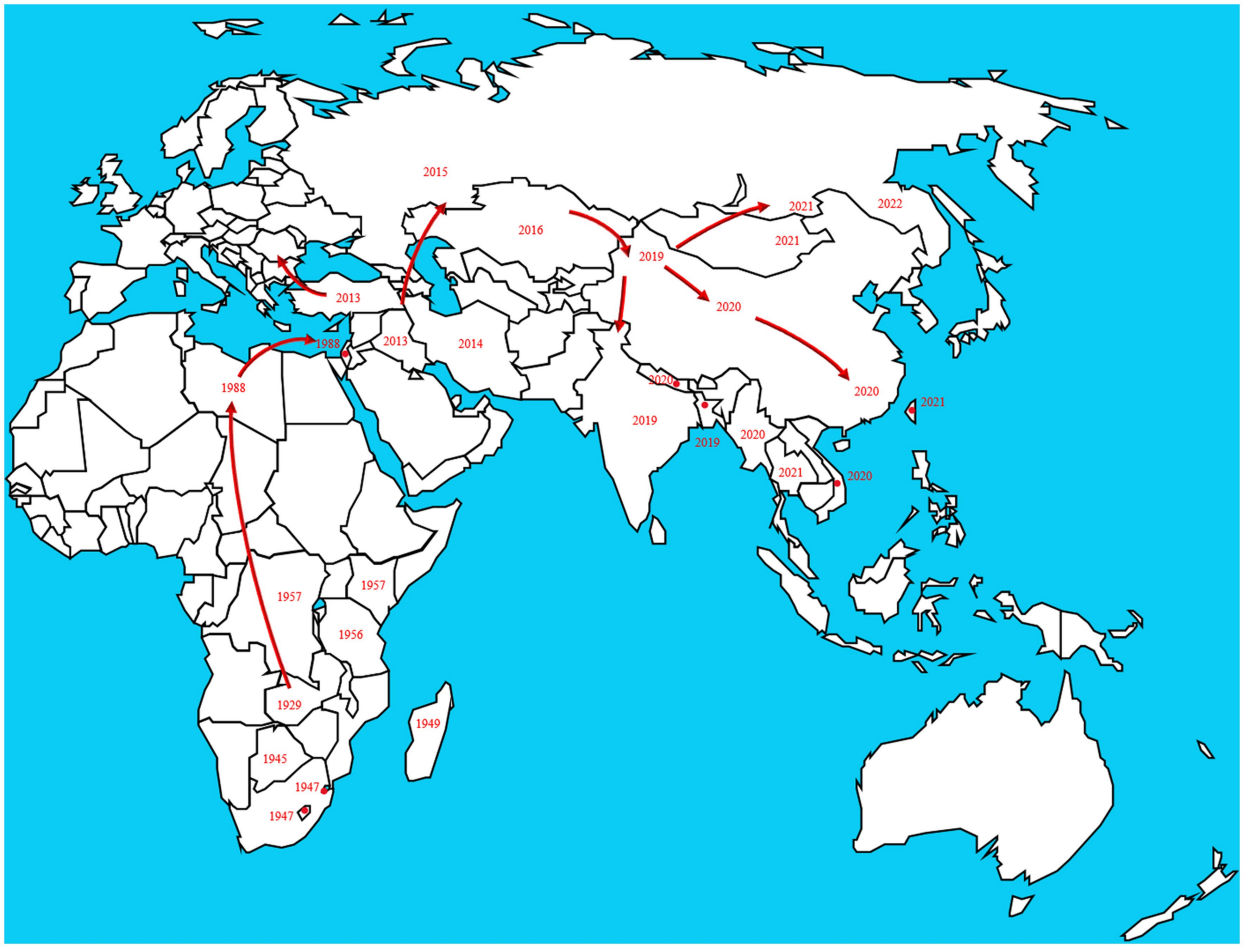
The disease originated in Africa and subsequently spread to European and Asian countries. The transmission route is based on the time and location of LSD which reported by the OIE in the past two decades. https://wahis.woah.org/#/dashboards/country-or-disease-dashboard/ (accessed 26 September 2022).

In 1929, LSD was found in the Zambia, Southern Africa, then it spread north to the Middle East (Weiss, 1968). By the 1940s, the disease had spread across the southern African countries, affecting plenty of livestock. During the following decades, LSD transferred slowly northwards, and it is currently present throughout virtually the entire African continent, including Madagascar. Libya, Algeria, Morocco and Tunisia are the only African countries unaffected by the disease. The first LSD outbreak in Egypt was reported in May 1988 (Ali et al., 1990). In 1989 there was an LSD outbreak in Israel (Yeruham et al., 1994). This outbreak was the first instance of LSD north of the Sahara Desert and outside of the African continent. After the year 2000, more and more outbreaks were reported by Middle Eastern countries and currently LSD is considered as an endemic disease in the region. At the end of 2013, the disease invaded into Turkey and Iraq. Incursion the disease into Iran and Azerbaijan was reported in the year after that. In the late 2014, the first LSDV cases were reported in the northern part of an island in the eastern Mediterranean, Cyprus. Turkey serves as an important crossroads connecting the Eurasian continent, facilitated the spread of LSD to the Balkans and some parts of the European countries. And it eventually spread to the northeast countries in Asia. Spread to the Russian Federation in 2015, followed by Kazakhstan in 2016. Then it was spread into Yi li, Xinjiang Province, China in 2019. In the following 2 years, the disease spread to southern and eastern parts of China and the countries in South Asia, including Nepal, Bhutan, Vietnam, Thailand and Myanmar (Pseudo-urticaria, 1931; Mulatu and Feyisa, 2018; Arjkumpa et al., 2021; Punyapornwithaya et al., 2022). By 2022, the disease had spread east and north to Mongolia and Eastern Siberia (Figure 2).

### Risk factors for LSD transmission

**Figure 3:**
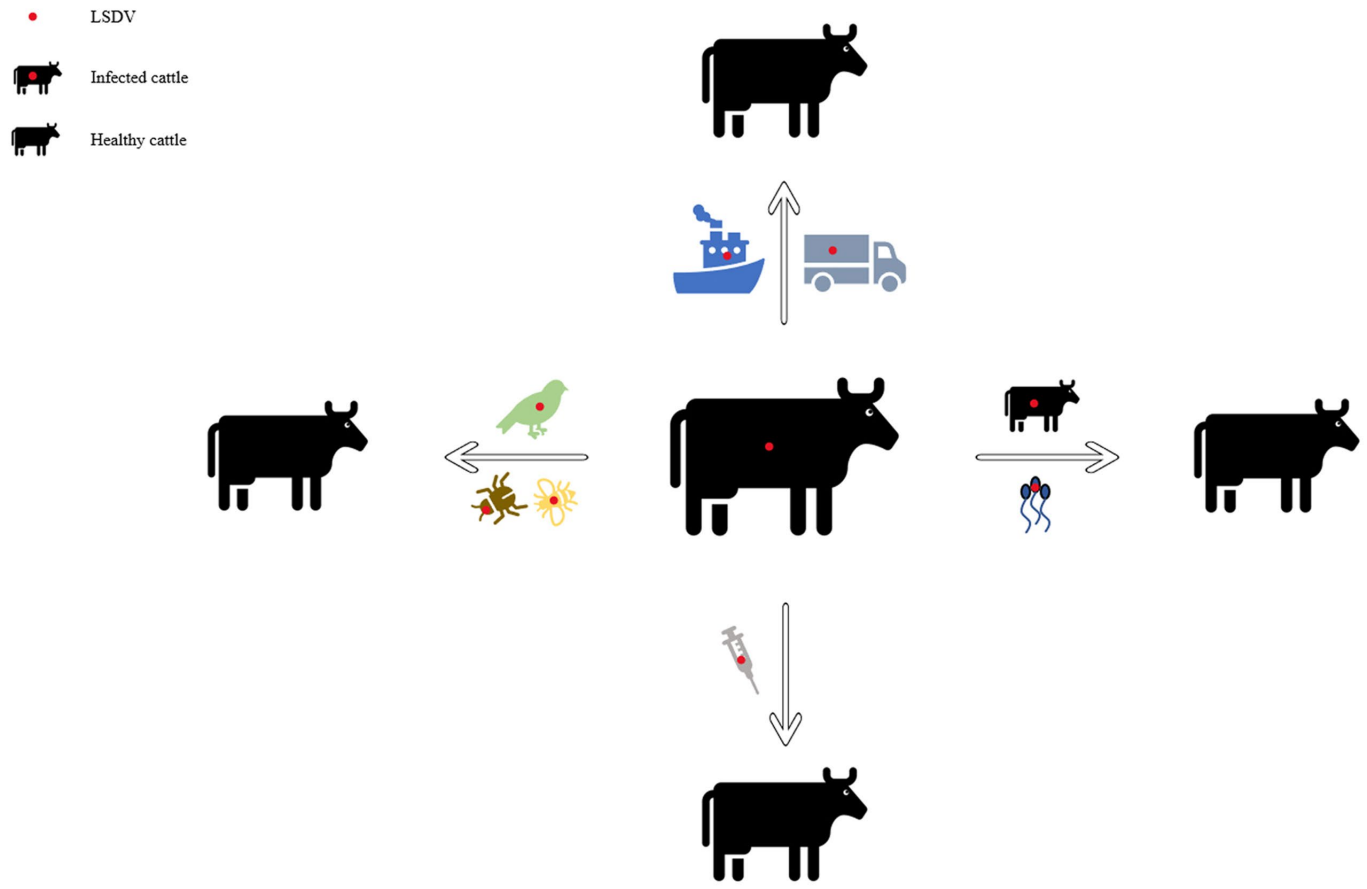
Diagram of the transmission modes of lumpy skin disease. This picture shows the propagation mode of LSDV more intuitively. Each infection mode corresponds to the one which was introduced in the article.

In early Africa, LSD may be widely spread due to long-distance migration of cattle. There is plenty of experimental data to support that LSDV is transmitted through the arthropods such as mosquitoes or midges, and the hematophagus such as hard ticks (Burdin and Prydie, 1959; Carn and Kitching, 1995; Tuppurainen et al., 2011; El-Ansary et al., 2022). The latter are the main vector of the virus. *Amblyomma hebraeum* ticks can transmit the virus by the mechanical/intrastadial and transstadial transmission modes (Lubinga et al., 2015). LSDV can live in *Aedes aegypti* female mosquitoes for a minimum of 2 to 8 days and infect other healthy cattle by themselves (Chihota et al., 2001; Sanz-Bernardo et al., 2021). In addition to the blood-borne virus transmitted by tick bites, it can also be taken by the female insects of *Amblyomma hebraeum* and *Rhipicephalus appendiculatus* passing through their eggs (Jongejan et al., 1980; Lubinga et al., 2014). The virus DNA can be detected in blood samples and nodular lesion area near the skin of susceptible cattle bitten by the larva of the *Rhipicehalus decoloratus* ticks. The larvae came from female ticks that had fed the blood from experimentally infected donors, and then the healthy experimentally cattle bitten by these small worms have mild symptoms typical of LSD (Tuppurainen et al., 2013). LSDV can protect itself from being destroyed by the wintering habits of the individual tick species (Bryson et al., 2002). LSDV can be transmitted by *Stomoxys calcitrans* and *Haematopota* spp., which are tiny blood-sucking insects (Sohier et al., 2019; Issimov et al., 2020). They are the most probably LSDV transmission vectors (Gubbins, 2019; Sanz-Bernardo et al., 2021). No direct studies have shown that LSDV can further multiply in vectors, but the basic reproductive number of LSDV in hosts varies greatly after the virus transmitted by different vectors reaches susceptible animals. *Stomoxys Calcitrans* has the highest reproductive number of 19.1, while *Aedes aegypti* has the lowest reproductive number of 2.4. That’s nearly eight-fold difference. However, it has been suggested that LSDV can survive *in vitro* culture of tick cell lines for 35 days without loss of infectivity (Tuppurainen et al., 2015). In addition to the bite of ticks, the bite of some species of mosquitoes can transmit the virus too. The latest British study confirmed that LSDV can exist in the mouthparts of four blood sucking insects including *Stomoxys calcitrans*, *Aedes aegypti*, *Culex quinquefasciatus*, and *Cubicoides nubeculosus*, for about 9 days, and then spread the disease by biting healthy cattle. This is the main way for mosquitoes to transmit LSDV (Sanz-Bernardo et al., 2022). According to early investigation in South Africa, it also can be spread by the direct contact, but at a significantly lower transmission rates and efficiency (Mulatu and Feyisa, 2018). Due to the limitation of tick’s mobility, flies, which are good at flying, have become one of the most harmful arthropod pests to the cattle worldwide (Gubbins, 2019). During the dry and cold seasons, the rate of this disease infection drops obviously, however, in the warm and wet period, the rate increases, which is closely related to the plummeting insect population and mobility. There is no significant association between sex or different cattle populations and seroprevalence of LSD infection. Furthermore, the variety of cattle, age, season, water supply and feeding system, introduction of breeding stock, and exposure to other species such as birds and insects all play important role in the occurrence of LSDV infection of LSD (Burdin and Prydie, 1959; Selim et al., 2021b). In addition to the direct contact and bites from blood-sucking insects, close-range transmission may also occur through LSDV-contaminated medical devices (Ali et al., 2012). Some viruses of the *Poxviridae* family can be transmitted through aerosols (Aleksandr et al., 2020). As a member of it, LSDV has also been reported that it may spread to other areas through air transport (Klausner et al., 2017). This is the cause of repeated LSD outbreaks in some countries and regions in the Middle East. It is also possible that the blood-sucking insects travel long distances with the help of air currents (Greenberg et al., 2012). But there is little chance of it spreading further into Russia and parts of Europe (Klausner et al., 2017; Saegerman et al., 2018). When sick cattle which carrying LSDV share a food tank with the healthy cattle to drink water or feed troughs, the healthy cattle will have typical symptoms of this disease (Ali et al., 2012). Researchers have pointed out that LSDV is difficult to spread through direct contact between cattle (Weiss, 1968; Carn and Kitching, 1995; Mulatu and Feyisa, 2018). Aleksandr et al. (2020) found that LSDV could be transmitted without the presence of flying insects and ticks. They speculated that it might be the contact between the skin and mucous membrane of healthy cattle and infected cattle that caused the transmission of the virus. The complexity of communication has not been fully analyzed, which can be used as a future research direction. Researchers has confirmed that it should be caused by the polluted snot and saliva of the sick livestock. The reason why the cattle with symptoms are different is that the virulence level of the virus is low, and the symptoms will be more severe if they come into contact with more viruses, while eating the food with less virus, they will show mild fever, the surface of skin does not even appear nodular lesions (Dietze et al., 2018). In another study, the experimenters reported that the viral loads in oral and nasal mucosa are comparable to those in skin nodules. The virus is most likely to be found in droplets and aerosols formed by the infected cattle and spread further by air currents. Therefore, saliva and nasal swabs can be a more convenient sampling method for the detection of this disease (Babiuk et al., 2008; Dietze et al., 2018). Even though experiments have shown that LSDV may be transmitted vertically from mother to offspring through the uterus (Şevik and Doğan, 2017). However, there were wounds on the surface of the born calf, which did not rule out the infection of the virus after birth. So the conclusion of vertical transmission needs to be verified (Rouby et al., 2016). Vaccinated cows could detect vaccine virus shedding in secreted milk (Bedeković et al., 2018). Therefore, vaccinated cows cannot breastfeed during the withdrawal period. An earlier study had confirmed that the LSDV in bovine semen for a long-term excretion. The experimental animals in this study, including azoospermic or severely oligozoospermic bull, can also detect the nucleic acid of LSDV by PCR, indicating that LSDV may exist in other body fluids than the semen fraction (Osuagwuh et al., 2007; Figure 3). Even if the clinical signs of the bulls are not obvious, they will continue to expel LSDV to the outside environment (Irons et al., 2005; Annandale et al., 2014). The testicles and lymph nodes of infected cattle can carry the virus, which can spread the disease. If these unsterilized animal products are transported over long distance by plane or truck, or live cattle with asymptomatic infection, or even infected cattle with obvious symptoms, as mentioned above, the disease can spread to other countries and regions (Kononov et al., 2019; Table 1). Recently, a new technology was developed to forecast the incidence of LSDV infection by assessing meteorological and geological attributes (Afshari Safavi, 2022). If this technology can be improved to predict and prevent the infection with antiparasitic drugs or vaccines in time, the losses from infection can be greatly reduced.

**Table 1:** The modes of transmission of lumpy skin disease and the summary of the countries and regions.

Routes of transmission	Susceptible animals	Countries and regions	References
Hard ticks	Cattle	Africa	Tuppurainen et al. (2011), Carn and Kitching (1995)
*Amblyomma hebraeum* ticks	Cattle	Africa	Lubinga et al. (2015)
*Rhipicehalus decoloratus* ticks	Heifer	South Africa	Tuppurainen et al. (2013)
*Aedes aegypti*	Cattle	North Africa	Chihota et al. (2001)
*Stomoxys calcitrans*	Cattle	Europe	Sohier et al. (2019)
*Haematopota spp*	Cattle	Europe	Sohier et al. (2019)
*Culex quinquefasciatus*	Calf	United Kingdom	Sanz-Bernardo et al. (2022)
*Culicoides nubeculosus*			
Birds	Cattle	Africa	Burdin and Prydie (1959)
Direct contact	Cattle	Africa and Asia	Aleksandr et al. (2020)
Vertical transmission	Calf	Turkey	Şevik and Doğan (2017)
Contaminated medical devices	Cattle	Africa	
Saliva and Nasal swabs	Cattle	Europe	Dietze et al. (2018)
Semen	Bull	South Africa	Osuagwuh et al. (2007)

### The effects of LSD on cattle health

The diseased cattle infected with LSD showed some clinical symptoms that could affect their health with naked eyes, such as edema of skin mucosa, decrease milk yield of cows, enlargement of lymph nodes, nodular lesions of different sizes on the skin surface, lameness of legs, etc. (Awad et al., 2010; Salib and Osman, 2011; Molla et al., 2017; Okur-Gumusova et al., 2020). The pathological changes of organs and tissues caused by LSDV infection in their bodies also affect their health.

Studies have shown that most of the organs and tissues of infected animals have pathological changes such as orchitis, cow mastitis, necrotic hepatitis, disseminated vasculitis, lymphadenitis, etc. A small number of cattle are accompanied by tracheitis, myocardial damage and other pathological changes, and can produce different intensity of injury induction in the affected animals, making LSDV more aggressive to the body (Ali et al., 2021). Ahmed et al. found in the clinical trial that the imbalance of oxidation antioxidation status in diseased cattle resulted in excessive increase of proinflammatory cytokine content and adverse effects on animals. Subsequently, the accumulation of metabolites in the liver, kidney and heart makes the organ function impaired, which leads to the occurrence of hypophosphatemia, and further aggravates the symptoms of hemolytic anemia (Ahmed and Dessouki, 2013). Abutarbush (2015) and Jalali et al. (2017) carried out the hematological and biochemical effect of LSD, the results showed that the blood of the affected cattle had pancytopenia, hyperproteinemia, hyperkalemia, hyperchloremia, and reduced creatinine concentration. It can be used as an index to evaluate the severity of the disease and to judge the prognosis.

Severe nodular lesions on the body surface of the cattle will cause holes in the skin, exposing the wound to the air. Affected cattle lack the effective protection of the first line of defense, and are prone to secondary infection with other bacterial or viral diseases, which may directly lead to their death. For susceptible cattle, timely prevention of disease and disinfection of diseased parts should be handled in place. Studies have shown that some nucleoside and nucleotide analogues can be used as anti-poxviruses drugs (De Clercq and Neyts, 2004). In the future, specific anti LSDV drugs should be developed. Under the premise of vaccination, drug assisted prevention will achieve better effects against the epidemic.

## The infective sensitivity of the host animals

As mentioned above, LSDV mainly infects cattle and water buffalo, but also has been reported to infect wildebeest, impala and giraffe (Young et al., 1970; Dao et al., 2022). After all, the number of infections is very small compared with the first two. As the main host animal of LSDV, cattle and buffalo have different susceptibility to this virus.

House et al. (1990) pointed out in an investigation report that all sheep, goats and buffaloes survived the LSD outbreak in Suez and Ismalia, showing no clinical symptoms. In recent years, there is also experimental evidence that buffaloes have low susceptibility to this virus (Neamat-Allah and Mahmoud, 2019). Researchers speculate that the reason is that buffalo have thick skin, and the mouthparts of blood sucking insects such as mosquitoes, flies and ticks cannot easily pass through the skin of buffalo, so the transmission rate and susceptibility of this disease are low (Chihota et al., 2003; Neamat-Allah and Mahmoud, 2019). It is also possible that because buffalo have been living in the pond for a long time, their skin is exposed to the air for a much shorter time than other breeds of cattle. This makes it difficult for blood sucking insects to touch their skin, resulting in a lower susceptibility to LSD. Barnard (1997) detected that there was no LSD antibody in wild buffaloes in South Africa, which may also indicate that buffaloes are not sensitive to LSD. However, the number of subjects is not large enough, the reliability of this study remains to be discussed. Some clinical trials also showed that buffalo inoculated with LSD vaccine could not effectively stimulate the body to produce anti LSD antibodies (Okur-Gumusova et al., 2020). This requires national veterinary authorities to timely and effectively assess the effectiveness of vaccines and develop vaccination strategies.

For cattle with strong resistance, such as buffalo, it may be able to resist the invasion of LSDV. The researchers said that this may be due to the insensitivity of buffalo to LSDV, which is only its non-adapted host. It may also be caused by the life habits of buffalo and the structure of their skin is different from that of ordinary domestic cattle. They also found that there was capripoxviruses in the buffaloes of the test group, which may be the symptom caused by other viruses rather than LSDV, making the laboratory staff not accurate enough to detect the content of LSDV antibody (Elhaig et al., 2017). Therefore, the future research direction should be to confirm the true pathogen in buffaloes with LSDV symptoms. If LSDV is indeed the culprit, scientists need to develop LSDV vaccines that specifically target buffalo and can induce high-level antibodies.

## Research advances in LSD vaccines

The prevention and elimination of infectious diseases ultimately depend on the largescale use of corresponding vaccines. Smallpox virus, a member of *Poxviridae* family that damages human life and health, has been eradicated in the last century after high-density mass inoculation of live vaccinia vaccines (Bhanuprakash et al., 2012). LSD is no exception as an infectious disease that seriously endangers the development of cattle industry (Wolff et al., 2020b). As far as the development of vaccines is concerned, it has roughly gone through live attenuated vaccines, inactivated vaccines, recombinant vaccines, combined vaccines, genetic engineering vaccines (Francis, 2018; Wang et al., 2020). The various vaccines that are developed by scientists over the years have their own characteristics, advantages and disadvantages.

### Live attenuated vaccines

Live attenuated vaccines, also known as attenuated vaccines, refer to natural virulent strains of microorganisms that have lost or weakened their pathogenicity to the original host animal through physical, chemical or biological treatment, and have been continuously passaged and screened. Vaccines prepared from strains that maintain good immunogenicity and genetic characteristics, or attenuated strains selected and multiplied from nature and culture conditions with good immunogenicity (Gershon et al., 1984). However, the live vaccine itself also has limitations, such as clinical side-effects, the risk of detoxification, and the risk of contracting new diseases due to homologous recombination with other viruses of this genus (Lee et al., 2012; Sprygin et al., 2018b; Krotova et al., 2022). Therefore, it is not recommended to be used in the areas without this disease. Immunosuppression is a factor to be considered after vaccination with attenuated vaccine, and its consequences can lead to a weak immune response to the vaccinated vaccine, while increasing the risk of secondary infection (Harland et al., 1992).

As the most representative strain of LSDV, the Neethling strain in South Africa was originally known as a virus similar to vaccinia virus, and it was the real pathogen that caused the outbreaks in Botswana in 1943 and South Africa in 1945 and then it was purified and named Neethling type (Stephens, 1966; Hunter and Wallace, 2001). Weiss (1968) investigated in clinical trials that the live vaccine made of this strain attenuated could play a certain prevention role against LSD. Then Weiss serially passaged this strain in the challocyst membrane of chicken embryos, resulting in the attenuation of the virus virulence. By the 20th passage, the virus did not cause systemic rash or other typical symptoms, and only half of the inoculated cattle localized swelling at the site that resolves within the next 4 to 6 weeks without signs of necrosis. Some mild side effects from this vaccine are called Neethling disease. The disease was also reported after vaccinating cattle with Neethling strain (Yeruham et al., 1994). Those vaccinated with the live attenuated Neethling strain produced a local response and the antibodies in the cattle were maintained for more than 3 years, and both cattle were resistant to the virulent strain even in cattle without a local response. Field study in Israel in 2012 concluded that Neethling had a lower incidence of morbidity after vaccination (Ben-Gera et al., 2015). The use of passaging and attenuation methods should be appropriate. If the virulence is excessively weakened, the immune effect will be counterproductive. The Neethling vaccine produced in Ethiopia could not protect vaccinated cattle against the virus challenge in clinical trials, with a protection rate of only 30% (Gari et al., 2015). One survey on a dairy farm in Northern Greece showed that after inoculation of adult cows with the Neethling strain, swelling was seen in 12% of immunized cows, which then subsided. Small skin nodules less than 0.5 cm in 9% of them, not in calves. Mild viremia occurs in vaccinated herds, luckily this condition is of short duration (Katsoulos et al., 2018). According to the (European Food Safety Authority, 2017), in Croatia, less than 1% of the cattle were vaccinated with Neethling vaccine and had adverse reactions (Calistri et al., 2019). It has been reported that Ethiopian Neethling vaccine was not protective against the disease (Gari et al., 2015). A study carried out by Bedeković et al. (2018) found that the vaccine virus could be detected in milk from cows vaccinated with this vaccine strain. Adverse reactions may occur when using the Neethling vaccine. Therefore, vaccine efficacy and safety should be fully evaluated to achieve the desired immune effect.

Haegeman et al. (2021) conducted numerous clinical trials and compared LSDV homologous live attenuated vaccines including Lumpy Skin Disease Vaccine (South-Africa), Lumpyvax (South-Africa), Kenyavac (South-Africa), Herbivac LS (South-Africa) and Vaccin LSD Neethling O vivant (Morocco). The above vaccines could cause the body to have a fever, but none negatively affected feed intake and daily activities and general health in all groups. Swollen lymph nodes in the group receiving the Herbivac LS vaccine. The remaining three vaccines from South Africa showed symptoms of Neethling disease after being vaccinated. Small nodules developed in the group vaccinated with the Moroccan Neethling vaccine, not as large as those found in infected animals (Haegeman et al., 2021). Considering the aforementioned Greek study, the subjects of the two experiments are very different, so there is a certain deviation in the data of clinical symptoms (Katsoulos et al., 2018). After the first three vaccines were inoculated and challenged by virulent strains, the virus can be detected in the blood. It is considered that the virus is detected in the blood after being challenged with the virulent strains, which is not the true viremia. None virus was detected in the blood of the cattle which were vaccinated with the latter two vaccines. The pathogen of contagious bovine pleuropneumonia (CBPP) is *Mycoplasma mycoides subsp. mycoides* (Mmm). Safini et al. (2022) made a bivalent vaccine by attenuated this pathogen (strain obtained from CIRAD AF262936) and Neethling strain (ID: AF409138), which can induce inoculated cattle to produce high-level neutralizing antibodies against the two diseases without clinical adverse reactions. It is predicted that the vaccine can protect the two diseases. However, there is no challenge with virulent strains, and the future test direction should be inclined to verify the protection after the challenge of virulent strains.

LSDV shares more than 97% nucleic acid sequence homology with GTPV and SPPV (Tulman et al., 2001, 2002). Therefore, cross-immunization of goat pox or sheep pox live attenuated vaccine is usually used clinically to prevent LSD. Back in the 1990s, veterinarians in Egypt controlled outbreaks of LSD using a vaccine against a Romanian poxvirus strain (Ali et al., 1990). Gari et al. (2015) verified that a sheep and goat pox (KSGP) 0–180 strain vaccines prepared in Kenya did not provide LSDV protection in cattle (Gari et al., 2015). Brenner et al. (2009) developed a clinical response after re-exposure to LSDV infection in Yugoslav RM65-vaccinated cattle during an epidemic in 2006–2007. Bamouh et al. (2021) demonstrated that the KSGP O-180 and KSGP O-240 vaccine strains may have the problem of vaccine virus shedding, thereby infecting other unvaccinated or other healthy cattle. The Gorgan goatpox vaccine (Gorgan vaccines) developed by the Jordan Biological Center was used to prevent goat pox virus in the Middle East in 2010 (Abbas, 2010), and then Gari et al. (2015) used this vaccine to study against LSDV and found that it can significantly stimulate the cellular immune response of vaccinated cattle, proved that the vaccine is highly immunogenic against LSDV. For two decades from 1989 to 2009, the Israeli authorities had used the RM-65 vaccine strain to control LSD and sheeppox, but the vaccine did not eliminate both diseases (Brenner et al., 1992; Yeruham et al., 1995).

When live vaccines are used to protect animals against viral and bacterial infections, the exact nature of the genetic transformation that results in attenuation is unknown. Since attenuating mutations occur randomly, a single point mutation that causes a virulence return in animals will occur. These uncontrollable factors make the attenuated vaccine a time bomb that can be detonated at any time (Minor et al., 1986). The previous description mentioned that the effects of some vaccine strains in the last century and this century were significantly different, which may be due to the base pair mutation of the vaccine strains during the production process. The incidence of homologous recombination of double-strand DNA viruses is high, and the vaccine may not exert its original effect due to the enhanced virulence of the virus after inoculation and other viruses of the *Poxviridae* family. Therefore, in the clinical application of live attenuated vaccines, specific problems should be analyzed in detail, and more suitable vaccines should be selected according to the actual situation of the cows to be vaccinated (Table 2).

**Table 2:** The live attenuated vaccines of LSD.

Strain	Virulence	Virus titer	Adverse reaction after vaccination	Challenge	Protection	Reference
South Africa “Neethling”	Low	10^4.5^ TCID_50_	50% of vaccinated cattle had swelling at the inoculation site	Do not verify	Do not verify	Weiss (1968)
Ethiopian “Neethling”	Low	10^3.5^ TCID_50_	No adverse reactions	Ethiopia LSDV-wild type	30%	Gari et al. (2015)
South Africa “Neethling” (Pirbright Institute)	Low	10^4.0^, 10^5.0^ TCID_50_	6.7% of vaccinated cattle showed Neethling disease	Do not verify	Do not verify	Bamouh et al. (2021)
KSGP O-180 (Kenyan sheep and goat pox)	Low	10^4.5^, 10^3.5^ TCID_50_	No adverse reactions	Ethiopia LSDV-wild type	50%	Gari et al. (2015)
KSGP O-240 (Kenyan (Kn) Sheep and Goat Pox by Pirbright Institute)	Low	10^4.0^, 10^5.0^ TCID_50_	3.7% of cattle vaccinated with low doses showed Neethling disease; 11.9% of cattle vaccinated with high doses developed skin lesions	Do not verify	Do not verify	Bamouh et al. (2021)
Gorgan GTP (Jordan Bio-Industries Centre (JOVAC))	Low	10^4.5^, 10^3.5^ TCID_50_	No adverse reactions	Ethiopia LSDV-wild type	100%	Gari et al. (2015)
South Africa Neethling (Onderstepoort Biological Products SOC Ltd.)	Low	10^3.5^ TCID_50_	12% of cattle developed lumps at the inoculation site; 9% of animals developed small lumps at the inoculation site after 8–18 days; Vaccine virus can be detected in milk from vaccinated cows	Do not verify	Do not verify	Katsoulos et al. (2018), Bedeković et al. (2018)
Homologous strain	Unknown	Unknown	0.09% of vaccinated animals developed fever, injection site edema, and decreased milk production	Do not verify	Do not verify	European Food Safety Authority, 2017
Lumpyvax (MSD Intervet South Africa (Pty) Ltd., Spartan, RSA, attenuated SIS type virus)	Low	10^4.0^ TCID_50_	Vaccine virus can be detected in milk from vaccinated cows	Do not verify	Do not verify	Bedeković et al. (2018)
Onderstepoort (Biological Products OBP; South Africa; batch 442)	Low	Unknown	86% of cattle showed hypothermia after vaccination	LSD/OA3-Ts.MORAN	100%	Haegeman et al. (2021)
Lumpyvax (MSD-Animal Health; South-Africa; batch BNDM07)	Low	Unknown	All cattle exhibited hypothermia after vaccination	LSD/OA3-Ts.MORAN	100%	Haegeman et al. (2021)
Kenyavac (Jordan Bioindustries Center Jovac; Jordan; batch 220,115–04)	Low	Unknown	71% of vaccinated cattle showed hypothermia after vaccination	LSD/OA3-Ts.MORAN	100%	Haegeman et al. (2021)
Herbivac LS (Deltamune; South-Africa)	Low	Unknown	Vaccinated cows had enlarged prethoracic lymph nodes; 57% of vaccinated cattle showed hypothermia after vaccination	LSD/OA3-Ts.MORAN	100%	Haegeman et al. (2021)
Vaccine LSD Neethling O vivant (MCI Santé Animal; Morocco, batch 17BLSDN001)	Low	Unknown	43% of vaccinated cattle had severe swelling greater than 10 cm in diameter at the inoculation site; 57% of vaccinated cattle showed hypothermia after vaccination	LSD/OA3-Ts.MORAN	100%	Haegeman et al. (2021)
RM65 (Abic Ltd. Netania, Isral)	Low	10^3.9^ TCID_50_	11.1% of the vaccinated cattle developed typical symptoms of LSD	Do not verify	Do not verify	Brenner et al. (2009)
combined Mmm/LSDV vaccine	Low	10^4.5^ TCID_50_ for LSDV 10^8^ CCU_50_ for Mmm	No adverse reactions	Do not verify	Do not verify	Safini et al. (2022)

### Inactivated vaccines

Inactivated vaccines refer to the inactivation of complete viruses (or bacteria) by physical, chemical and biological methods, so that the pathogens are sufficiently killed, infectivity or virulence is lost, and their immunogenicity is maintained. It has the advantages of low production cost, short development cycle and good usage effect. Compared with live attenuated vaccines, inactivated vaccines usually require booster immunization to prevent virus invasion (Bhanuprakash et al., 2004). Blackall (1988) reported in 1988 that the use of vaccine adjuvants can effectively enhance the effect of inactivated vaccines.

There are no reports of inactivated LSD vaccines circulating on the market. It was found that the use of Bi-ethylimine bromure (BEI) to inactivate the attenuated Neethling strain also provided good protection. A variety of antibodies can be detected and the virus neutralization test demonstrated that the antibody response rate of the inactivated vaccine was 37% higher than that of the live attenuated vaccine on the 28th day after vaccination (Hamdi et al., 2020). Y Es-sadeqy et al. developed a bivalent inactivated vaccine with oil adjuvants against the LSDV and Bluetongue virus (BTV) in 2020, and stimulated the production of high levels of neutralizing antibodies considered animals welfare and animal ethics, no challenge test to be conducted, so the specific clinical effect needs to be verified by further experiments (Es-Sadeqy et al., 2021). Wolff et al. (2020b) pointed out that different vaccine adjuvants can make inactivated vaccines more effective. The use of adjuvant A, whose main component is a low molecular weight copolymer, and adjuvant B is composed of amphotericin, Quil A and cholesterol, both adjuvants were used in the inactivated Neethling vaccine and Serbia vaccine have no adverse reactions. Adjuvant A can effectively stimulate the humoral immune response and the production of IFN-γ in vaccinated cattle, so it has become a clinically preferred adjuvant. Matsiela et al. (2022) inactivated Neethling strain with low concentration of BEI, used Montanide™ Gel-01 as vaccine adjuvant, and immunized rabbits to obtain a high level of neutralizing antibody. It has not been tested in cattle, and the new adjuvant developed can be used as a reference.

After the inactivated vaccine is injected into the animal, other proteins and antigen components in it will also induce the body to react. Therefore, in addition to interfering with the host’s immune response, it will also induce unwanted immune responses, which facilitates the antigen extraction process, optimizations and improvements (Stephens et al., 1984). One disadvantage of inactivated vaccines is that they generate a narrow immune spectrum, although they are excellent in inducing humoral immunity, they are rarely effective in inducing cell-mediated immune responses and mucosal immune responses. Therefore, it is still necessary to prepare a safer and more efficient inactivated vaccine against LSDV (Table 3).

**Table 3:** The inactive vaccines of LSD.

Strain	Virus titer	Adjuvant	Adverse reaction	Challenge	Protection	Reference
South Africa “Neethling”	10^6.0^ TCID_50_	Oil	No adverse reactions	Virulent LSDV Israeli field isolate	100%	Hamdi et al. (2020)
LSDV-BTV4	10^6.0^ TCID_50_	Oil	No adverse reactions	BTV4	LSDV do not verify; BTV 100%	Es-sadeqy et al. (2021)
LSDV- “Neethling Vaccine”	10^7.0^CCID_50_	Low molecular weight copolymers	No adverse reactions	LSDV “Macedonia2016” field strain	100%	Wolff et al. (2020b)
LSDV- “Serbia” field strain	10^6.0^CCID_50_; 10^7.0^CCID_50_	A combination of Amphigen, Quil A and Cholesterol	Presents as mild Neethling disease	LSDV “Macedonia2016” field strain	100%	Wolff et al. (2020b)
OBP- “Neethling Strain	10^6.0^TCID_50_	Montanide™ Gel-01	–	–	–	Matsiela et al. (2022)

### Recombinant vaccines

Because the live attenuated vaccine can keep all relevant antigens in the vaccine, and the pathogen can replicate in the host, it can stimulate the host’s cellular immunity and humoral immunity, so it is considered to be the most ideal method. Unfortunately, traditional methods cannot attenuate all pathogens. As mentioned above, even if the virus is attenuated, virulence return may occur. In order to overcome these problems, some scientists had tried to identify the virulence genes of different pathogens, change the virulence of pathogens by directional mutation or deletion of these virulence genes, and achieve attenuated strain in a recombination way (Liang et al., 1991).

Deletion of the thymidine kinase and glycoprotein genes of herpes virus did not change its normal replication *in vitro* (Kitching et al., 1987). Then Romero et al. pointed out that the recombinant capripox virus vaccine expressing the rinderpest fusion protein gene was prepared by using homologous recombination to knock out the thymidine kinase gene of pox virus and then recombine with the fusion protein gene of rinderpest virus, which can effectively protect vaccinated cows from rinderpest and the threat of lumpy skin disease (Romero et al., 1993). Ten years later, Ngichabe et al. (2002) confirmed the reliability of the recombinant vaccine for rinderpest and goat pox through successive research results. A single vaccination protected cattle from rinderpest and lumpy skin disease virus for up to a year and the cattle in other experimental group can be protected for up to 3 years. Johnston and McFadden (2003) found that poxviruses can encode a homologue of interferon gamma to competitively block the binding of interferon gamma produced in the host to its natural receptor, thereby achieving the purpose of immune escape. Wendy et al. (2002) found that the aphthous virus gene can encode the production of interleukin-10-like, which subsequently produces immunosuppressive effects on host cells. On this basis, D. Kara et al. used homologous recombination technology to construct deletion of LSDV open reading frames 005 and 008, and then constructed recombinant vaccines, including LSDV-WB005KO and LSDV-WB008KO. Clinical trials after that, the aggregation of the two can effectively stimulate the neutralizing antibody level of the vaccinated cattle, which can effectively resist the invasion of LSDV. However, in the early stage of vaccination, there will be clinical reactions that are small and can be subsided (Kara et al., 2018). It was previously reported that LSDV-WB005KO also protected vaccinated animals from SPPV and GTPV (Boshra et al., 2015). Then the LSDV-WB005KO may be a better choice in clinical practice.

Recombinant poxviruses, like other vaccines, are concerned by regulatory authorities that vaccines made from recombinant viruses will also be released into the environment, posing a safety hazard to healthy animals. The solution to this problem is to study suicidal or non-replicating recombinant viruses. Graham and Prevec (1992) developed replication-deficient adenoviruses. These viruses can replicate *in vitro* in cells containing the E1 region, but cannot replicate in normal cells. Even if healthy animals are exposed to the virus, it is safe. In the following 2 years, Heffner and Peeters et al. reported that some herpes viruses must delete their glycoprotein genes to replicate in cell lines containing glycoprotein genes, but not in normal cells. The development in a direction of safe use also provides a new idea for the preparation of vaccines after recombination of LSDV with other viruses (Table 4; Heffner et al., 1993; Peeters et al., 1994).

**Table 4:** The recombinant vaccines of LSD.

Vector strain	Insert genes	Delete genes	Challenge	Protection rate	References
Kenya sheep-1(KS-1)	Fusion(F) protein gene of RPV	–	Virulent lumpy skin disease virus	100%	Romero et al. (1993)
			Saudi 1/81 RPV strain	100%	
Kenya sheep-1(KS-1)	Haemaglutinin and fusion protein genes of RPV	–	Virulent Neethling strain	100%	Ngichabe et al. (2002)
			Kabete O strain	55%	
LSD OBP vaccine (ht-LSD-OBP)	–	Interleukin-10-like (IL-10)	LSDV-WB	100%	Kara et al. (2018)
LSD OBP vaccine (ht-LSD-OBP)	–	Interferon gamma recptor-like (IFN-γR)	LSDV-WB	100%	

### Vaccine carrier

Therapeutics that deliver DNA into the body to express the corresponding protein in some way is a long-term goal that will require the efforts of generations. Miller and Dusty (1992). published an article saying that from more difficult techniques such as transplantation of transfected cells (lymphocytes, myoblasts, hematopoietic stem cells), to more direct methods, such as the use of viral vectors (reverse transcription Viruses, adenoviruses, herpesviruses, parvoviruses) deliver DNA to target tissues in the body, thereby stimulating the activation of the body’s immune system. Poxviruses have been widely studied as vaccine vectors. Due to their large genome, vary in size from 130 to 375 kb (Holowczak, 1982). Such innate conditions allow them to tolerate the insertion of foreign genes of more than 25,000 base pairs. In the presence of a highly active promoter, the simultaneous expression of multiple exogenous genes can be achieved, and the humoral immunity and cellular immunity can be effectively activated (Zavala et al., 2001; Willey et al., 2003). In addition, it has a narrow host spectrum and is safe as a vaccine carrier (Hunter and Wallace, 2001). The virus is heat-resistant, which can reduce the cost of refrigerated storage. At the same time, a multivalent vaccine can prevent multiple diseases after injection, which is far safer and more cost-effective than multiple injections of a single vaccine to achieve the same effect of preventing multiple diseases (Prow et al., 2018).

Aspden et al. (2002) recombined the glycoprotein gene of rabies virus into LSDV as a vector (rLSDV-RG), this recombinant virus can stimulate the humoral immune response after clinical trials, the results show that 75% of cattle can resist rabies virus significantly stronger than the control group. Subsequently, the same author in 2003 published a paper confirming that the vaccination of non-ruminant animals such as rabbits and mice with this vaccine induce rabies virus-neutralizing antibodies twice as high as reported by the World Health Organization (WHO). The level of neutralizing antibodies produced after vaccination of the mice was comparable to that of the commercially available rabies vaccine (Aspden et al., 2003). Wallace and Viljoen (2005) found that two viral recombinants were constructed by using lumpy skin disease virus as a vector and the thymidine kinase gene located on it as the insertion site of foreign genes. They are the structural glycoprotein gene expressing Bovine Epizootic fever (LSDV-BEFV) and the two genes expressing Rift Valley fever glycoprotein (LSDV-RVFV). These two virus recombinants can induce the production of neutralizing antibodies in experimental animals. After LSDV-BEFV stimulation test, high levels of neutralizing antibodies can be stimulated. Mice inoculated with LSDV-RVFV are resistant to RVFV up to 100%.

The genes recombined with LSDV listed above are all from RNA viruses (Rabies virus, Bovine Epizootic fever virus, Rift Valley fever virus), so this other multivalent vaccine brings new ideas. At the same time, it also provides a reference for the development of vaccines for lumpy skin disease.

## Research advances on the diagnostic methods of LSD

The on-site diagnosis of LSD often relies on clinical symptoms to determine whether the cattle are infected with the disease. However, in the early stages of the disease, affected cattle often show only fever and very few skin lesions, which greatly reduces the accuracy of the diagnosis. According to the researchers’ findings, the clinical symptoms of lumpy skin disease are similar to those of bovine herpesvirus infection, demodicosis, bovine viral diarrhea-mucosal disease and bovine malignant catarrhal fever (Reid et al., 1984; Deregt and Loewen, 1995; Reddy and Sivajothi, 2016). The presence of these factors complicates field diagnosis. Therefore, more accurate detection methods such as directly targeting the pathogen in the laboratory are needed (Perry, 2010). Sometimes after vaccination, animals still suffer from the disease. In consequence, judging whether animals were infected with the more virulent wild strain or the vaccine strain adversely affected the animals. Then it is particularly important to distinguish whether there is a wild virus or a vaccine strain in the animal (Chibssa et al., 2018; Fawzi et al., 2022).

### Diagnostic methods of The LSD

Heine et al. (1999) found that SPPV and LSDV in the genus *Capripoxvirus* have a P32 gene with a nucleic acid sequence similarity of more than 98%. Two nucleotide site mutations in the P32 gene of LSDV lead to two *Eco*R V sites are missing. Therefore, the researchers used this idea for clinical testing, using specific primers B68 and B69 to amplify the P32 gene by polymerase chain reaction (PCR) in the viral samples collected from the field, followed by restriction endonuclease digestion susceptibility to determine whether the cattle infected with LSDV or SPPV. If this feature is used as a diagnostic method, more *Capripoxviruses* need to be sequenced to confirm that the *Eco*R V locus is specific for all SPPV. Babiuk et al. found that the presence of LSDV could be detected using real-time PCR. Compared with the oral and nasal mucosa, the detection rate of the diseased material in the skin nodule injury site was higher. This provides a reference for the collection of clinical patient materials in the future (Babiuk et al., 2008). In October of the same year, Stram et al. (2008) reported that they analyzed the non-homologous sequences of the three viruses (GTPV, SPPV and LSDV) in the genus *Capripoxvirus*, found a nucleic acid sequence that only exists in LSDV, and then designed PCR primers for this sequence to distinguish LSDV. A comparative experiment by Awad et al. (2010) in October of the following year showed that the detection rate of virus in the blood of infected cow skin biopsy specimens and infected animals by PCR can reach 100%, and the detection rate of virus in the blood of febrile cows can reach 77.8%. All were significantly higher than the virus detection rate of virus isolation, dot blot hybridization and indirect enzyme-linked immunosorbent assay (ELISA). Therefore, PCR method can be used as a fast and efficient tool for LSDV field infection diagnosis (Awad et al., 2010). In 2016, a portable, simple, and rapid method called recombinase polymerase amplification (RPA) assay for the field detection of the genome of LSDV (Shalaby et al., 2016). Yana et al. (2018) published a technique based on real-time high-resolution fusing PCR. The nucleic acid sequence of the viruses (SPPV, GTPV, LSDV) in the disease materials collected on site was amplified by PCR, and then the three viruses were distinguished according to the melting temperature of the generated amplicons. The gene that specifically targets LSDV is LSDV-ORF010, which has the unique species-specific nucleotide differences. Subsequently, Modise et al. (2021) found on this basis that the type of virus can be analyzed by the high-resolution melting (HRM) assay of PCR amplification products generated after genus-specific primers amplify sample viral DNA and bind dyes. Some farms do not have expensive and high-precision instruments, such as PCR machines, and the staff that on the farm may not have the skills to operate PCR machines, so a cheaper, convenient, reliable and easy-to-operate method is needed to replace PCR. Mwanandota et al. (2018) explored a novel method for the detection of LSDV, named loop-mediated isothermal amplification (LAMP). This method target the poly (A) polymerase small subunit (VP39) gene because of its higher detection rate and sensitivity. It is possible to detect extremely small amounts of nucleic acid substances present in the air with experimental error. Zeedan et al. (2019) compared PCR, real-time PCR (qPCR), fluorescent antibody technology (FAT), indirect FAT (iFAT) and indirect ELISA (iELISA) for the detection of LSDV and the positive rate of LSDV antibodies. In the skin disease material detection group, the detection rate of qPCR was better than that of conventional PCR, which could reach 39.13%. The virus detection rate of the FAT method was the lowest at 32.6%. All were higher than the antibody positive rate detected in blood. For the detection of LSDV antibodies in serum, iELISA was 3.45 percentage points higher than iFAT. This also confirms what was mentioned above, suggesting that when collecting disease materials on the spot, the skin nodule lesion area is preferentially collected as a test sample (Zeedan et al., 2019). Then, Haegeman et al. (2020) developed a novel high-sensitivity, high-specificity assay, the Immunoperoxidase Monolayer Assay (IPMA), which can detect LSDV antibodies early in vaccination and disease infection. This technology can be used in simple and crude environment detection, with high safety and can be processed in ordinary biosafety level laboratory. The same year, a novel study was reported by Milovanovic et al. (2020) using ELISA to detect LSDV-specific antibodies in milk. The advantage of this new technology is non-invasive sampling, which can collect a wide range of samples and can be used for large-scale screening. Sequence differences in three genes, RPO30, P32, and GPCR, were analyzed using single-nucleotide polymorphism using nanopore sequencing technology to build a database to distinguish GTPV, SPPV, and LSDV. The ease of replication of this database makes it more widely used. The advantages of this technology are that it is suitable for complex clinical environments, short detection time, and strong portability (Eltom et al., 2021). Lesions caused by LSDV in other species can also be used as a differential diagnosis method, for example, the isolation of the virus into embryonated chicken eggs (ECEs) can cause characteristic pitting lesions on the chorioallantoic membrane (El-Ansary et al., 2022). The pathological sections made from the skin lesions can be diagnosed by immunohistochemical (IHC) methods, and the distribution of pathogenic antigens can be detected by specific anti-LSDV antibodies. Changes in the dermis and epidermis of the skin after infection with the virus can be observed under the microscope, including watery degeneration, granulomatous reaction, dystrophic calcification of the dermis, and the formation of inflammatory cells (El-Neweshy et al., 2013; Sanz-Bernardo et al., 2020; Amin et al., 2021). Ali et al. observed inclusion bodies in the cytoplasm of bovine skin capsule through histopathological examination, and confirmed that these inclusion bodies were characteristic pathological lesions related to LSD. In recent years, some researchers confirmed this view in clinical tests (Ali et al., 1990; Ahmed and Dessouki, 2013; Neamat-Allah and Mahmoud, 2019). A new rapid diagnostic technique for LSDV-ORF068 gene targeting using recombinase polymerase amplification assay (RPA) combined with CRISPR-Cas12a-based fluorescence assay (RPA-Cas12a-fluorescence assay). It can be detected in trace amounts with excellent accuracy and sensitivity. There is no cross-reactivity with other common bovine viruses (Jiang et al., 2022). A rapid diagnostic tool of colorimetric sandwich-type lateral flow immunoassay (LFIA) was established using two monoclonal antibodies against different epitopes of P32 structural protein of LSDV and gold nanoparticles (Cavalera et al., 2022). The sensitivity of this new method is similar to that of ELISA, but it has not been widely used in clinical diagnosis, and its specificity needs to be determined after clinical trials. All the above methods require instruments and power equipment to detect. Korthase et al. (2022) invented a method of extracting nucleic acid without electricity, namely Triple*E*, which can extract nucleic acid from 8 samples within 10 min and ensure sensitivity. It can be applied to the place without good experimental conditions for diagnosis. For the diagnostic methods of detecting a specific gene to determine the type of virus, we need to screen a large number of viral nucleic acid types to ensure that the above-mentioned specific gene is not in the local affected cows with similar symptoms due to the homologous recombination or gene mutation (Table 5).

**Table 5:** The diagnostic methods of LSDV.

Method	Targets	Accuracy	Reference
Polymerase chain reaction (PCR)	P32 gene	98%	Heine et al. (1999)
	LSDV nucleic acid	Skin samples 100%	Awad et al. (2010)
		Blood samples 77.8%	
	LSDV nucleic acid	Skin samples 34.78%	Zeedan et al. (2019)
		Blood samples 28.26%	
Real-time PCR (qPCR)	ORF074 gene	Unknown	Babiuk et al. (2008)
	LSDV nucleic acid	Skin samples 39.13%	Zeedan et al. (2019)
		Blood samples 36.95%	
Pathological section examination	Intracytoplasmic inclusion bodies	Unknown	Ali et al. (1990)
Recombinase polymerase amplification (RPA) assay	LSDV nucleic acid	100%	Shalaby et al. (2016)
Real-time high-resolution fusing PCR	LSDV-ORF010	Unknown	Yana et al. (2018)
Loop-mediated isothermal amplification (LAMP)	VP39 gene	68.42%	Mwanandota et al. (2018)
Fluorescent antibody technique (FAT)	LSDV protein	Skin samples 26.08%	Zeedan et al. (2019)
		Blood samples 32.60%	
Indirect Enzyme-linked immunosorbent assay (iELISA)	LSDV antibodies	17.93%	Zeedan et al. (2019)
Indirect FAT (iFAT)	LSDV antibodies	14.48%	Zeedan et al. (2019)
Immunoperoxidase monolayer assay (IPMA)	LSDV antibodies	100%	Haegeman et al. (2020)
Enzyme-linked immunosorbent assay (ELISA)	LSDV-specific antibodies in milk	Unknown	Milovanovic et al. (2020)
High-resolution melting (HRM)	PCR amplicons of samples	Unknown	Modise et al. (2021)
Nanopore sequencing	RPO30, P32 and GPCR	Unknown	Eltom et al. (2021)
Histopathological examination	Skin pathology section	Unknown	Sanz-Bernardo et al. (2020), Ali et al. (2021)
Trials for VI and identification on ECEs	LSDV	Unknown	Ali et al. (2021)
Immunohistochemical (IHC)	LSDV antigen	Unknown	Ali et al. (2021)
RPA-Cas12a-fluorescence assay	ORF068 gene	96.3%	Jiang et al. (2022)
Lateral flow immunoassay (LFIA)	P32 gene	Unknown	Cavalera et al. (2022)
Triple*E*	LSDV nucleic acid	Unknown	Korthase et al. (2022)

Different diagnostic methods can be selected according to the stage of the disease in clinical application. In the early stage of LSDV infection, when the clinical symptoms are not obvious, some highly sensitive detection methods can be used, such as nucleic acid level, antigen antibody level detection methods. Early diagnosis, detection and treatment can effectively prevent and treat in advance. When the clinical symptoms obviously, the characteristic pathological changes can be used for differential diagnosis.

### Distinguish between wild-type and vaccine viruses of LSDV

Menasherow et al. (2014) found three methods to detect vaccine strains versus wild strains. First, the Neethling vaccine strain is 27 bases shorter than the Israeli virulent strain of the enveloped virions (EEV) gene, which can be distinguished by genetic sequencing based on this finding. The second method is to use primers to amplify the genome of the virus, and then use specific upstream and downstream primers to amplify by nested PCR, and determine the composition of the virus according to the difference in annealing temperature. The last method is to digest the amplicon of the PCR reaction according to the Mbo I enzyme cleavage site. The vaccine strain samples can be digested by Mbo I enzyme, but the virulent strain cannot be digested. Clinically, the second and third identification methods are more rapidly and widely used (Menasherow et al., 2014). Agianniotaki et al. (2017) developed a dual real-time PCR method in 2017, targeting GPCR genes, and the amplification efficiency of wild-type virus and the vaccine virus was 91.3% and 90.7%. According to the amplification efficiency of the sample to identify whether it is a wild-type virus or a vaccine virus. Several detection methods that have been researched and discovered so far need to analyze the obtained data to get the results. Möller et al. (2019) developed a new technology based on TaqMan probe-based independent double-stranded real-time qPCR (real-time quantitative PCR) detection method to distinguish virulent strains from vaccine strains with 100% analytical sensitivity and specificity. Also targeting the EEV gene, Agianniotaki et al. (2021) developed a novel surveillance tool, duplex real-time PCR, to specifically detect the presence of wild-type LSDV in samples containing high titer vaccine against LSDV. In the presence and absence of LSDV vaccine virus, the amplification efficiencies of virus samples were 99.0% and 98.6%. The experimenters set β-actin as an internal amplification control to increase the detection accuracy (Table 6).

**Table 6:** The methods of distinguishing the vaccine and wild-type viruses of LSDV.

Method	Target molecule	Accuracy	Reference
Nucleic acid sequence detection	Enveloped virions (EEV) gene	Unknown	Menasherow et al. (2014)
Duplex real-time PCR		Unknown	Agianniotaki et al. (2021)
Nested PCR	Genome of virus	Unknown	Menasherow et al. (2014)
PCR	MboI enzyme cleavage site	Unknown	Menasherow et al. (2014)
Dual real-time PCR	GPCR genes	Unknown	Agianniotaki et al. (2017)
Double-stranded real-time qPCR	TaqMan probe	100%	Möller et al. (2019)

## Conclusion

Prevention and control should be carried out according to the local disease transmission mechanism and the living habits of species that are often used as transmission vectors. Eliminate those species in the right season to reduce their exposure to susceptible animals. Although there is substantial evidence of widespread vector-borne disease, LSD also spreads during periods when insects are inactive. Studies by Klausner et al. (2017) have reported that the virus may be transmitted through the atmosphere with aerosols, but it is not completely certain. Therefore, further investigation of the role of airflow on the transmission of LSDV is required. After the epidemic, there may be problems such as immune recessive infection and continuous detoxification, and it is necessary to further clarify and standardize the treatment of the same herd of cattle. High temperature fumigation can also effectively avoid the breeding of LSDV and eliminate the hotbed of virus.

For remote areas, it may not be financially feasible to fully vaccinate and eradicate disease, but vaccinated cattle should be permanently marked at least. These vaccinated and non-vaccinated cattle can be managed together, and using herd immunity to reduce the losses from outbreaks. At present, live attenuated vaccines are more commonly used. However, such live vaccines have the risk of homologous recombination with other viruses, causing the vaccinated animals to be infected with other new viruses. An inactivated vaccine targeting LSDV has not yet been developed. Researchers have developed a live attenuated vaccine that can be inactivated and used with adjuvants to achieve a good preventive effect, but it has not been widely used in clinical practice (Hamdi et al., 2020). For countries and regions without this disease, the problem of inactivated vaccines still needs to be solved urgently. There are no reports on subunit vaccines with higher safety profile. LSDV has been used in the laboratory as a vector for recombinant subunit vaccines for some diseases (Aspden et al., 2002; Wallace and Viljoen, 2005). It has been studied that the H3L gene of vaccinia virus is the main immunodominant envelope protein of the mature virus in cells, which can induce the production of neutralizing antibodies (Kumar et al., 2017). In addition, the LSDV14 gene is similar to the K3L gene of vaccinia virus and the M156 gene of myxoma virus, which inhibit the phosphorylation of protein kinases, thereby inhibiting the production of interferon (Beattie et al., 1995; Peng et al., 2016). These may prompt the production of safe and efficient vaccines in other ways. In order to overcome the risk of virulence reversion and homologous recombination of attenuated vaccines, the development of safer and more efficient recombinant vaccines may be the direction of LSDV vaccine development in the future. By inserting a gene at a specific location in the LSDV genome, it can replicate in a specific cell line, but not in the host, resulting in higher safety (Klupp et al., 1992; Heffner et al., 1993). Therefore, it is necessary to deeply study the pathogenic mechanism of LSDV, and explore the virulence genes and immunosuppressive genes, so that the pathogen can be weakened to different degrees through targeted mutation or deletion. Studies have shown that ivermectin (IVM) can inhibit the viral titer of LSDV and attenuate the transmission of LSDV (Toker et al., 2022). As an insect-borne disease, LSDV can be targeted to study whether other anti-parasitic drugs can be used in conjunction with vaccines to effectively block the spread of LSDV.

Currently used diagnostic methods with highly sensitivity, such as LAMP and RPA-Cas12a-fluorescence assay, are all targeting viral genes. However, these detection methods are easily affected by aerosol contamination, which affects the accuracy of the diagnostic results (Liu S. et al., 2021). Therefore, future research should focus on how to overcome the pollution caused by these aerosols.

Since 1929, LSD has endangered the healthy development of the global cattle industry for nearly a 100 years. Scientists are doing everything they can to eliminate this pathogen. The feasible prevention and diagnosis methods that have been developed need to be verified by a large number of clinical trials. Therefore, cutting off effective transmission routes, large-scale safe and efficient use of vaccines, and correct detection methods are the directions of efforts in the future. However, we still do not have a deep understanding of the pathogenic mechanism of this disease. This review highlighted the current research progress of this disease, and puts forward prospects for the insufficiency of research and future research trends, providing information for the elimination of the disease.

## Author contributions

ZL, YS, XY and XW conceived and designed the study. ZL, KY, SW, JY, XM, YS, XY wrote the manuscript. All authors listed have made a substantial, direct, and intellectual contribution to the work and approved it for publication.

## Funding

This study was supported by the grants from the Key Development and Research Foundation of Gansu (grant no. 21YF5WA153), Natural Science Foundation of Gansu Province (21JR7RA022), and Natural Science Foundation Project of China (grant no. 32202779).

## Conflict of interest

The authors declare that the research was conducted in the absence of any commercial or financial relationships that could be construed as a potential conflict of interest.

## Publisher’s note

All claims expressed in this article are solely those of the authors and do not necessarily represent those of their affiliated organizations, or those of the publisher, the editors and the reviewers. Any product that may be evaluated in this article, or claim that may be made by its manufacturer, is not guaranteed or endorsed by the publisher.
